# Essential Role of Cyclophilin A for Hepatitis C Virus Replication and Virus Production and Possible Link to Polyprotein Cleavage Kinetics

**DOI:** 10.1371/journal.ppat.1000546

**Published:** 2009-08-14

**Authors:** Artur Kaul, Sarah Stauffer, Carola Berger, Thomas Pertel, Jennifer Schmitt, Stephanie Kallis, Margarita Zayas Lopez, Volker Lohmann, Jeremy Luban, Ralf Bartenschlager

**Affiliations:** 1 Department of Molecular Virology, University of Heidelberg, Heidelberg, Germany; 2 Department of Microbiology and Molecular Medicine, University of Geneva, Geneva, Switzerland; The Rockefeller University, United States of America

## Abstract

Viruses are obligate intracellular parasites and therefore their replication completely depends on host cell factors. In case of the hepatitis C virus (HCV), a positive-strand RNA virus that in the majority of infections establishes persistence, cyclophilins are considered to play an important role in RNA replication. Subsequent to the observation that cyclosporines, known to sequester cyclophilins by direct binding, profoundly block HCV replication in cultured human hepatoma cells, conflicting results were obtained as to the particular cyclophilin (Cyp) required for viral RNA replication and the underlying possible mode of action. By using a set of cell lines with stable knock-down of CypA or CypB, we demonstrate in the present work that replication of subgenomic HCV replicons of different genotypes is reduced by CypA depletion up to 1,000-fold whereas knock-down of CypB had no effect. Inhibition of replication was rescued by over-expression of wild type CypA, but not by a mutant lacking isomerase activity. Replication of JFH1-derived full length genomes was even more sensitive to CypA depletion as compared to subgenomic replicons and virus production was completely blocked. These results argue that CypA may target an additional viral factor outside of the minimal replicase contributing to RNA amplification and assembly, presumably nonstructural protein 2. By selecting for resistance against the cyclosporine analogue DEBIO-025 that targets CypA in a dose-dependent manner, we identified two mutations (V2440A and V2440L) close to the cleavage site between nonstructural protein 5A and the RNA-dependent RNA polymerase in nonstructural protein 5B that slow down cleavage kinetics at this site and reduce CypA dependence of viral replication. Further amino acid substitutions at the same cleavage site accelerating processing increase CypA dependence. Our results thus identify an unexpected correlation between HCV polyprotein processing and CypA dependence of HCV replication.

## Author Summary

Owing to limited genetic information, viruses have to exploit host cells to achieve efficient production of virus progeny. Host cell factors and pathways therefore play an important role for virus replication and thus represent a possible target for antiviral therapy. In case of the hepatitis C virus (HCV), an RNA virus infecting liver cells and causing chronic liver disease, host cell cyclophilins were shown to play an important role in replication. Pharmacological inhibition of cyclophilins, which are catalysts of protein folding, causes profound inhibition of HCV replication, but neither the underlying mechanism by which cyclophilins contribute to viral replication, nor the exact nature of the cyclophilin are known. In this study we demonstrate that HCV replication and presumably also virus particle assembly requires cyclophilin A (CypA), which can be blocked by the cyclosporine analogue DEBIO-025. We identify mutations affecting proteolytic cleavage of the viral polyprotein that render HCV replication less dependent on CypA and thus cause DEBIO-025 resistance. Studies with additional mutants reveal a correlation between polyprotein cleavage kinetics and CypA dependence. Our results support a model by which CypA activates the viral replicase in a manner that depends on the kinetics with which the viral polyprotein is cleaved.

## Introduction

The hepatitis C virus (HCV) is a hepatotropic virus that has a high propensity to establish persistence. At present, more than 170 million people suffer from chronic hepatitis C [1]. Current therapy of this disease is based on the combination of pegylated interferon-alpha and ribavirin. However, sustained viral response rates are not satisfying and side-effects associated with this therapy are high. Thus, the development of alternative and more effective strategies to counteract HCV infection are of great importance. One promising drug showing potent anti HCV function is cyclosporine A (CsA). The cyclic undecapeptide CsA is a secondary metabolite of the fungus *Tolypocladium inflatum* and was originally discovered as powerful immunosuppressive drug [2]. The immunosuppressive properties of CsA are due to its ability to block the phosphatase Calcineurin in activated T cells [3] by binding with high affinity to cyclophilins (CyPs) [4]. Because of the non-immunosuppressive properties combined with profound antiviral activity, CsA derivatives such as DEBIO-025 [5], NIM811 [6], and SCY-635 are more likely to be used as anti HCV agents. Unlike CsA, these molecules binds to CyPs but do not display calcineurin inhibition.

As a member of the *Flaviviridae* family, HCV has a positive strand RNA genome encoding a single polyprotein that is cleaved by cellular and viral proteases into 10 different proteins (reviewed in [7],[8]). The structural proteins core, envelope protein 1 (E1) and E2 reside in the N-terminal region of the polyprotein and they are the major constituents of the virus particle. Virus assembly and release requires p7, a presumed viroporin [9]–[11] and nonstructural protein 2 (NS2) that contains a complex N-terminal trans-membrane domain and a C-terminal protease domain responsible for cleavage between NS2 and NS3 [12],[13]. The latter is composed of two domains, an N-terminal protease domain which is activated via the NS4A cofactor and a C-terminal helicase domain. NS4B most likely plays a major role in the induction of membrane alterations that are required for the assembly of viral replication complexes. NS5A is an RNA binding protein required for replication and virus assembly [14]–[16] and NS5B is the RNA-dependent RNA polymerase (RdRp). Cleavage of the polyprotein in the NS3 to NS5B region is mediated by the NS3/4A protease complex. Processing at the NS3-4A site is a rapid intramolecular reaction. Subsequent cleavages take place intermolecularly in the following preferred, but not obligatory order: NS5A-B, NS4A-B, NS4B-5A [17],[18]. Replication of HCV occurs in the cytoplasm of infected cells in distinct virus-induced compartments designated the membranous web [19]. It is a complex membrane network composed of an accumulation of membranous vesicles of various sizes and interspersed lipid droplets which are the presumed sites of HCV assembly [16], [20]–[22]. It is thought that core and NS5A are key players in mediating transfer of viral proteins and RNA from viral replication complexes to lipid droplets to trigger assembly [16],[21],[22].

Our knowledge about cellular proteins required for HCV RNA replication and virus assembly is scarce, but recent studies suggest that cyclophilins (Cyp) play an important role. Cyps are molecular chaperones catalyzing the cis-trans isomerization of proline residues and hence are called peptidyl-prolyl cis-trans-isomerases (PPIases). Up to now 16 Cyp members have been identified with 7 major members found in humans (CypA-E, Cyp40, CypNK; [23]). Cyps share a common domain, but other than their PPIase activity differ in subcellular distribution and function. Conflicting data exist as to which Cyp is required for HCV replication. Watashi and coworkers reported that CypB is important for viral replication and they observed a direct binding of CypB to NS5B which results in enhanced RNA binding and thus increased RNA polymerase activity [24]. Others reported that CypA or CypA, B and C are required for HCV replication [25],[26]. For both CypA [26] and CypB [24],[27],[28] direct interactions with NS5B were reported arguing for a direct involvement of cyclophilins in HCV RNA replication. However, the underlying mechanism is not known.

We report here that an enzymatically active CypA is essential for HCV replication and describe an interrelation between CypA requirement for HCV replication and processing kinetics at the NS5A-B site.

## Results

### CypA but not CypB is essential for HCV replication and virus production

**Figure 1:**
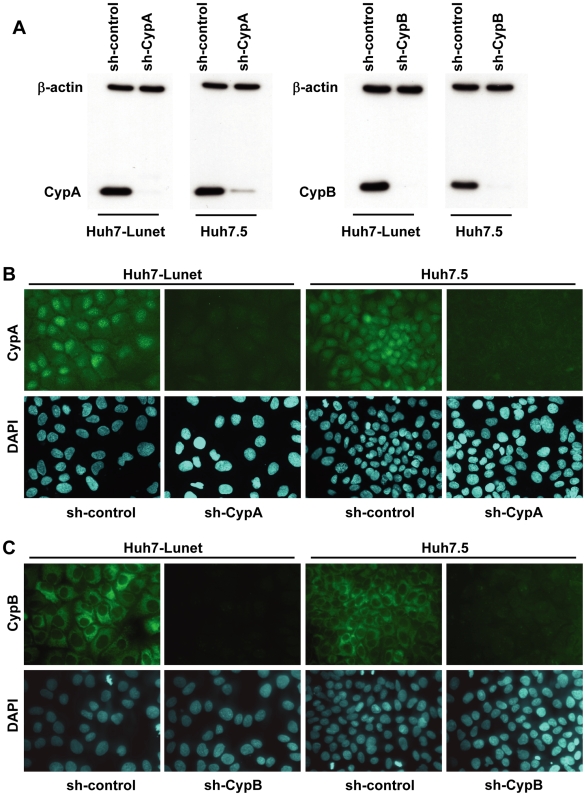
Major role of CypA for HCV replication. (A–C) Stable knock-down of CypA (left panel) or CypB (right panel) in Huh7.5 and Huh7-Lunet cells was achieved by retroviral transduction of shRNAs targeting the 3′ NTR of either mRNA. As a reference, cells were transduced with the retroviral vector encoding an unrelated shRNA sequence (sh-control). (A) Knock-down efficiency was determined by western blot with a CypA or a CypB specific antibody. Beta-actin was used as loading control. (B, C) Detection of CypA (B) and CypB (C) knock-down in Huh7-Lunet and Huh7.5 cells via indirect immunofluorescence analysis. Cells were seeded onto glass cover slips and fixed with methanol 72 h later. Immunostaining was performed with CypA or CypB specific antibodies.

Contrary opinions about which type of Cyp is essential for HCV prevail in current literature [24],[26]. To clarify these contradictions we established a panel of cell lines derived from the highly permissive Huh-7 subclones Huh7.5 and Huh7-Lunet by retroviral transduction of shRNAs targeting the 3′ non-coding region of CypA or CypB (Fig. 1). Cell pools transduced with the retroviral vector expressing an unrelated shRNA were generated as a control. Transduced shRNAs lead to a profound and stable reduction of the expression of CypA or CypB in the respective cell pools as detected by Western blot (Fig. 1A) and immunofluorescence analysis (Fig. 1B–C).

**Figure 2:**
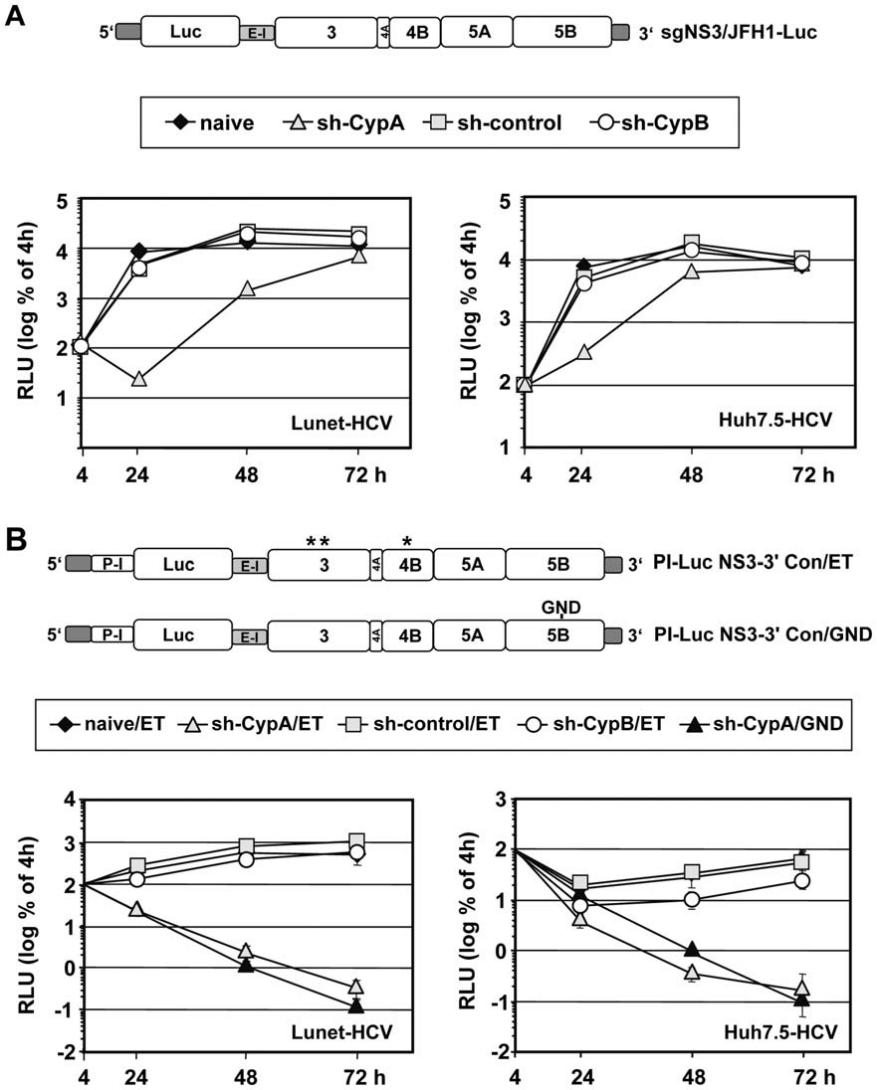
Impact of stable CypA or CypB knock-down on RNA replication of genotype 2a and 1b replicons. (A) Huh7-Lunet cells (left panel) and Huh7.5 cells (right panel) either stably transduced with the unrelated shRNA control (sh-control) or a vector encoding the CypA- or CypB-specific shRNA or naive cells were transfected with a subgenomic luciferase reporter replicon derived from the HCV isolate JFH1 (sgNS3/JFH1-Luc, shown at the top). Cells were lysed at given time points after transfection, and luciferase activity (expressed in relative light units, RLU) in cell lysates was determined. Values are normalized to the 4 h value that reflects transfection efficiency and that was set to 100%. Means and standard deviations of a representative experiment are shown. Luc, firefly luciferase; sg, subgenomic replicon. (B) Impact of CypA or CypB knock-down on replication of Con1. Schematic diagrams of the two Con1-derived constructs are shown in the top. They are composed of the HCV 5′ NTR, the IRES of poliovirus for high-level expression of the firefly luciferase gene, the EMCV IRES, the HCV replicase coding region and the 3′ NTR. To enhance RNA replication, 3 cell culture adaptive mutations (labelled with asterisks) have been introduced in case of the wild type (ET) replicon [31]. The GND mutant contains an active site mutation destroying NS5B RdRp activity, thus serving as control to measure decay kinetics of luciferase activity expressed from transfected input RNA. Huh7-Lunet cells (left panel) and Huh7.5 cells (right panel) specified below the schematics of the replicons were transfected and replication was analyzed as described in the legend of panel A. One experiment of two independent repetitions, each performed in duplicate is shown.

To determine the impact of reduced CypA or CypB expression on HCV RNA replication, cells were transfected with a subgenomic JFH1 luciferase reporter replicon RNA (sgNS3/JFH1-Luc; Fig. 2A, upper panel). Cells were lysed 4, 24, 48 and 72 h after transfection and replication was scored by measuring luciferase activity in cell lysates. Luciferase activity was normalized to the 4 h value, reflecting transfection efficiency. Replication was not affected by CypB knock-down and was comparable between naïve and vector control-transduced Huh7-Lunet or Huh7.5 cells (Fig. 2A, left and right panel, respectively). However, when we transfected the same replicon into cells with the stable CypA knock-down luciferase activity was reduced more than 100-fold at the early time point (24 h post transfection) in case of Huh7-Lunet cells and about 50-fold in case of Huh7.5 cells. Only at the latest time point (72 h post transfection) luciferase activities were comparable in all transfected cell lines. This kinetic is due to the fact that replication of JFH1 is limited by the host cell [29]. For this reason RNA replication phenotypes are often only detectable at early time points after transfection before host cell factor(s) (other than CypA) become limiting and thus restrict HCV replication in naïve cells. Therefore, HCV replication in cells with the CypA knock-down can ‘catch up’ until it reaches wild type level [30]. By using the luciferase values (which because of the very short half life of luciferase are ideally suited to determine replication kinetics) we calculated the apparent doubling time of HCV RNA during the exponential phase in the various cell lines. We found that apparent doubling time in naïve and control cells were in the range of 3.7 h whereas apparent doubling time was 11.4 h in Huh7-Lunet cells with stable CypA knock-down. In case of Huh7.5-derived cell lines apparent doubling times were 3.6 h and 7.4 h for control and CypA knock-down cells, respectively. Thus results obtained with both cell lines were remarkably similar arguing against a cell line-specific phenotype.

Given the controversial discussion on genotypic differences with respect to Cyp-dependence of HCV replication, we also determined the impact of CypA and CypB knock-down on replicons derived from the genotype 1b isolate Con1. As shown in Fig. 2B, replication of a highly adapted Con1-derived replicon (Con/ET; [31]) was completely blocked in Lunet cells with CypA knock-down as deduced from the comparable drop of luciferase expression in cells transfected with this replicon or the replication defective NS5B active site mutant (Con/GND). In contrast, replication of Con1/ET was completely unaffected by knock-down of CypB expression. In case of Huh7.5 derived cell lines that support Con1 RNA replication to a much lower extent, we also observed a complete inhibition of replication in CypA knock-down cells, but in some experiments a slight inhibition in cells with a CypB knock-down was found as well (Fig. 2B, right panel). The latter was however, at the limit of statistical significance (Mann-Whitney-Test).

Finally, we studied whether CypA dependence of HCV replication is specific for this virus or applies to other flaviviruses by analyzing replication of a Dengue virus 2 (New Guinea C isolate) replicon in the same cell lines. We found that RNA replication of Dengue virus was not affected by CypA or CypB knock-down in Huh7-Lunet cells (Fig. S1, left panel) whereas in Huh7.5 cells with a CypA knock-down a slight reduction of replication was found (right panel). However, also in this case this difference was at the limit of statistical significance. In summary, we concluded that CypA is an essential host cell factor required for RNA replication of HCV but not Dengue virus, whereas CypB appears to play no role for HCV replication.

**Figure 3:**
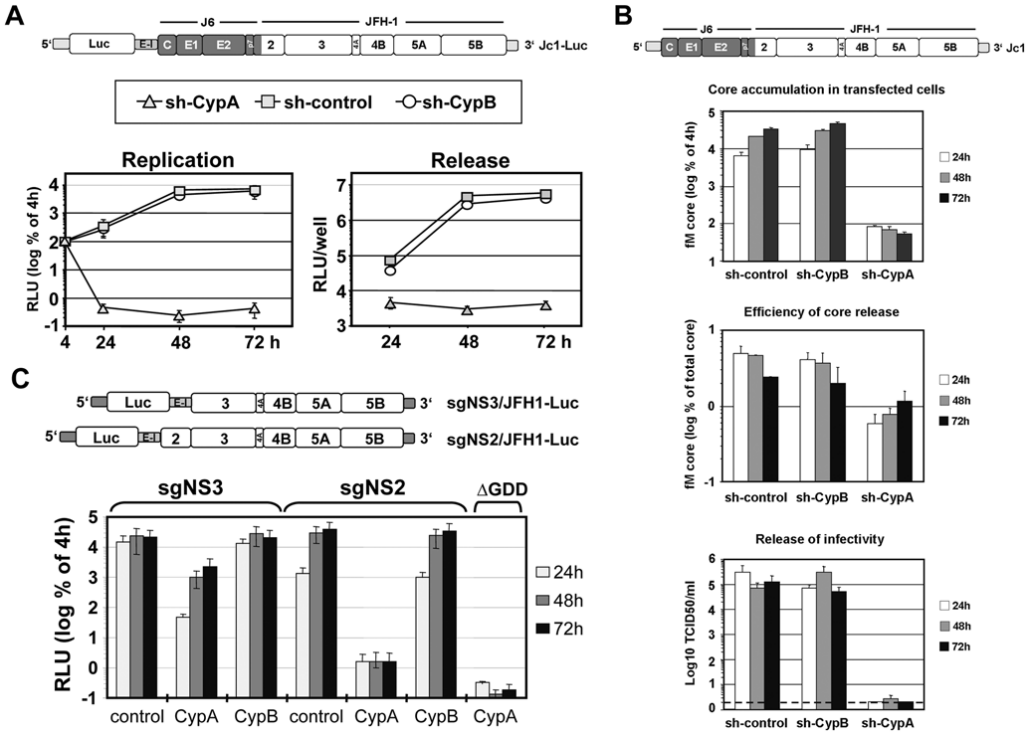
Increased inhibition of RNA replication of JFH1 full length genomes and NS2-containing replicons by CypA knock-down. (A) Huh7-Lunet cells stably transduced with the control vector (sh-control) or the vector encoding the CypA- or CypB-specific shRNA were transfected with the Jc1-Luc genome (shown at the top). Replication of the genome was determined by luciferase assay with cell lysates prepared at given time points (left panel). Infectivity released from transfected cells was measured by inoculation of naive Huh7.5 cells with culture supernatants harvested at given time points after transfection and luciferase activity was determined in infected cells 72 h after inoculation (right panel). Means and standard deviations of two independent experiments are shown. The dark shaded region in the HCV genome map in the top indicates the J6-derived sequence; the white region corresponds to JFH1. (B) Huh7-Lunet cells were transfected with the Jc1 genome and accumulation of intracellular core protein amounts was determined by core-specific ELISA (upper panel). Kinetic of core release is shown in the middle panel. Core protein in culture supernatants of transfected cells was normalized to total core protein amounts (intra- plus extracellular core). Kinetic of release of infectious virus particles is shown in the bottom panel. Infectivity was determined by TCID_50_ assay. Background of the assay is indicated by the horizontal dashed line. (C) Huh7-Lunet cells were transfected with JFH1-derived subgenomic replicons encoding an NS3 to 5B or NS2 to 5B replicase of JFH1 (sgNS3/JFH1-Luc and sgNS2/JFH1-Luc, respectively) and replication was determined by luciferase assay as described in the legend to panel (A). Background of the assay was determined with the sgNS3-derived replicon with an active site mutation destroying NS5B RdRp activity (ΔGDD). A representative experiment of three independent repetitions, each performed in duplicate is shown.

To study the impact of these knock-downs on replication of full length HCV genomes we used a bicistronic reporter genome (Jc1-Luc; Fig. 3A, upper panel). It is derived from the highly assembly competent chimera Jc1 composed of the genotype 2a isolate J6 and JFH1 that are fused at a distinct site in the N-terminal trans-membrane domain of NS2 [32] (Fig. 3A). For this and all subsequent studies phenotypic analyses were performed primarily in Huh7-Lunet cells because of the more potent knock-down of CypA and CypB expression as compared to Huh7.5-derived cell lines (Fig. 1). A very potent inhibition of Jc1-Luc replication was found in CypA, but not CypB knock-down cells (Fig. 3A, lower left panel). In fact, the inhibition was much stronger compared to the one observed with the subgenome and replication did not recover even at later time points. Infectivity was not detected in supernatants of CypA knock-down cells (Fig. 3A, right panel), but not significantly reduced in supernatants of CypB knock-down cells.

The profound inhibition of Jc1-Luc replication in CypA knock-down cells might be due to the less robust replication of this bicistronic genome compared with the sgNS3/JFH1-Luc replicon [33]. Alternatively, CypA dependence of JFH1 full length HCV genomes and subgenomes may differ. We addressed these possibilities by transfection of CypA knock-down cells with a full length genome lacking heterologous sequences. Owing to highest assembly efficiency we used the Jc1 chimera (Fig. 3B). Viral replication was scored by determination of intracellular core protein amounts whereas virus production was determined by measurement of core released into the supernatant of transfected cells as well as infectivity titers in culture supernatant (TCID_50_; see materials and methods). Replication of the Jc1 genome was potently reduced in CypA knock-down cells, but unaffected by CypB knock-down. In addition to impaired replication, core release was also reduced as deduced from the relative core protein amounts in cell lysate and culture supernatant. In agreement with this profound block of replication and impaired assembly no infectivity was detected in supernatants of CypA knock-down cells even though virus titers in control cells were in the range of 10^5^ TCID_50_/ml and higher.

The congruent results obtained with the genomic reporter replicon and the Jc1 genome suggest that CypA targets an additional viral factor outside of the minimal replicase (NS3 to NS5B). We assumed that NS2 would be the most likely candidate for three reasons. First, cleavage at the NS2-3 site by NS2 is essential for RNA replication [34]; second, the structural proteins core, E1 and E2 as well as p7 are dispensable for RNA replication [35]; third, NS2 is required for HCV assembly [12],[13]. To put this assumption to the test we performed comparative transient replication assays by using subgenomic JFH1 replicons encoding either an NS2 to NS5B or NS3 to NS5B replicase (sgNS2/JFH1-Luc and sgNS3/JFH1-Luc, respectively) (Fig. 3C). Replication of the sgNS2 replicon was almost completely blocked in Huh7-Lunet cells with a CypA knock-down and only slightly above background as determined with the replication defective NS5B active site mutant JFH1/ΔGDD. In contrast, replication kinetic of the sgNS2 RNA was well comparable between Huh7-Lunet cells with a CypB knock-down and control cells that had been transduced with a non-targeting luciferase shRNA. We note a lower replication of the sgNS2 replicon in CypB knock-down cells at 24 h post transfection as compared to the sgNS3 replicon, but this reduction was also observed with control cells (Fig. 3C). At later time points, replication of sgNS2 and sgNS3 replicons was comparable. In summary these results provide genetic evidence that CypA also targets a factor outside of the replicase, most likely NS2.

### Isomerase activity of CypA is essential for HCV RNA replication

**Figure 4:**
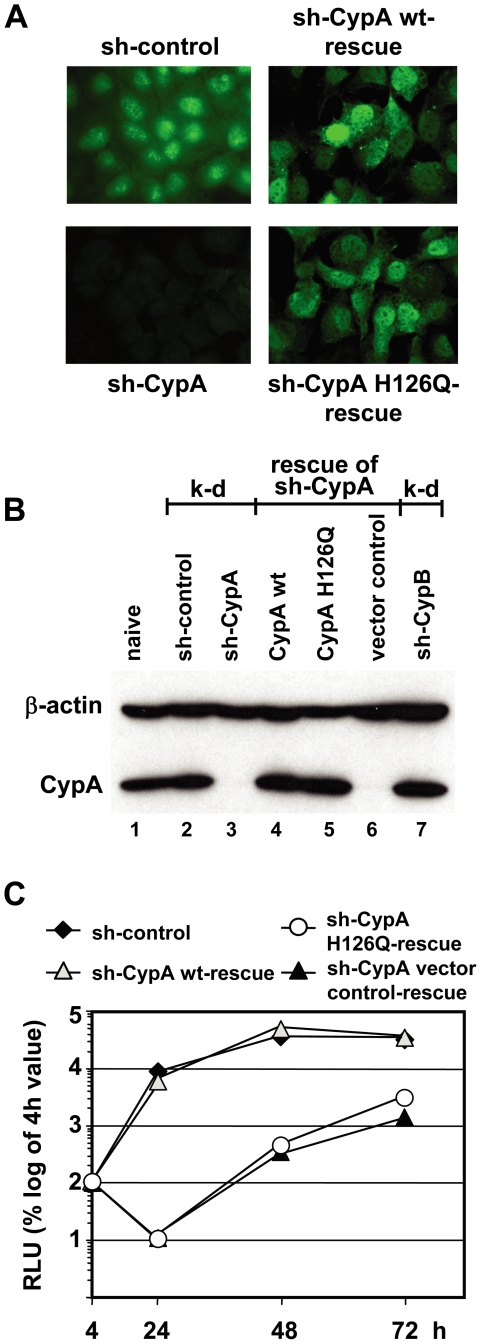
Rescue of HCV replication by overexpression of CypA wt but not CypA H126Q in stable knock-down cells. (A) Huh7-Lunet cells with a stable knock-down of CypA (lower left) were stably transduced with a CypA wild type (wt) expression construct (CypA wt-rescue; upper right) or a CypA expression construct carrying the H126Q mutation (CypA H126Q-rescue; lower right) that are both resistant to the shRNA. For comparison, Huh7-Lunet cells stably transduced with the same expression vector containing the non-related control shRNA (sh-control) are shown (upper left). Cells were analyzed by immunofluorescence using a CypA-specific antibody. Note that CypA expression levels are higher in the ‘rescue cells’ as compared to the vector control cells (sh-control). (B) Lysates from cells specified in the top were analyzed by Western blot using a CypA-specific antibody. Lane 6 shows a Western blot of a lysate of Huh7-Lunet cells with a stable knock-down of CypA and transduced with an empty vector (cell line sh-CypA vector control-rescue). Beta-actin was used as a loading control. K-d, knock-down; rescue, stable CypA knock-down cells transduced with CypA wt or the H126Q mutant or empty expression vector, respectively. Loaded samples are specified above each lane. (C) Restoration of HCV replication by ectopic expression of CypA wt, but not CypA H126Q in stable CypA knock-down cells. Huh7-Lunet cells specified in the top were transfected with a bicistronic JFH1 reporter replicon (sgNS3/JFH1-Luc) and RNA replication was determined by luciferase assay 24, 48 and 72 h post transfection. Values are normalized for transfection efficiency by using the 4 h value. Means and standard deviations of a representative experiment of two independent repetitions are shown.

Although the results described so far clearly support an important role of CypA for HCV replication, we could not exclude that the observed phenotype was due, at least in part, to off-target effects. For this reason, we transduced the Huh7-Lunet CypA knock-down cell lines with a CypA expression construct that was not targeted by the shRNA (‘rescue’ cell line in Fig. 4). In addition, we generated CypA knock-down cell lines expressing a CypA mutant (H126Q) that retains less than 1% of wild type isomerase activity [36],[37]. As shown in Fig. 4A and B, CypA expression levels achieved upon transduction of the shRNA-resistant CypA genes was very high (Fig. 4A). In fact, by using quantitative immunofluorescence we found that expression level of CypA in wild type ‘rescue’ cell lines was about two-fold higher as compared to sh-control cells (234+/−4.7 counts per cell based on 8-bit gray value histograms, generated with the ImageJ software package, versus 125+/−25.3 counts per cell, respectively) see materials and methods). Knock-down of CypA had no effect on expression levels of CypB (Fig. 4B, lane 7) and vice versa (data not shown). Upon transfection of CypA wt ‘rescue’ cells with sgNS3/JFH1-Luc, RNA replication was fully restored (Fig. 4C) thus excluding off-target effects of the shRNA that was used for silencing of CypA expression. However, no rescue was obtained in cells stably transduced with the H126Q mutant (Fig. 4C), arguing that isomerase activity of CypA is essential for HCV RNA replication.

### CypA is the target for antiviral activity of the cyclosporine analogue DEBIO-025

**Figure 5:**
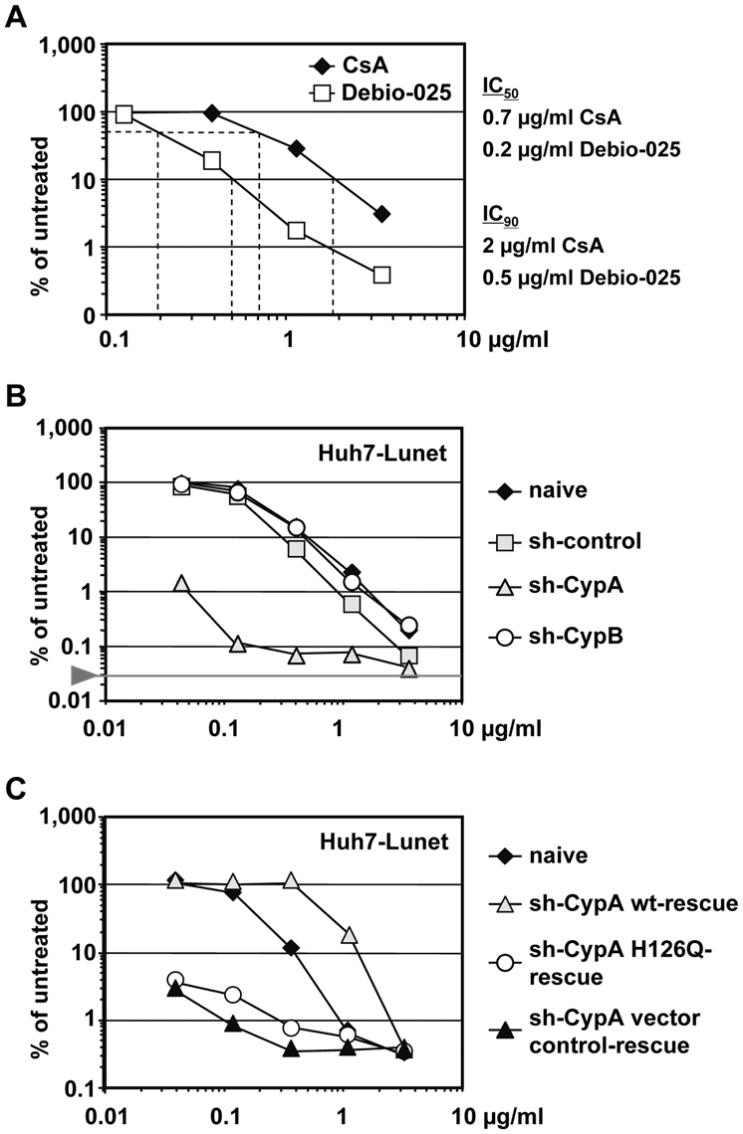
CypA is the target of the cyclosporine analogue DEBIO-025. (A) Dose-response curve for cyclosporine A (CsA) and DEBIO-025. Huh7-Lunet cells transfected with JFH1 reporter replicon (sgNS3/JFH1-Luc, shown in the top of Fig. 2A) were incubated 4 h post electroporation with escalating concentrations of CsA or DEBIO-025 and 72 h later cells were lysed and luciferase activity was determined. (B) Naive Huh7-Lunet cells or cells transduced with a retroviral vector encoding a CypA- or CypB-specific shRNA or transduced with the retroviral vector control (sh-control) were transfected with a HCV luciferase reporter replicon (sgNS3/JFH1-Luc). Four hours post transfection various concentrations of CsA or DEBIO-025 were added, cells were incubated for 72 h and lysed. Luciferase activities were determined and normalized to the values obtained with mock-treated cells, which was set to 100%. The background of the assay is indicated with the grey horizontal line. (C) Huh7-Lunet cells with a stable knock-down of CypA and transduced with the empty vector (control-rescue) or the shRNA-resistant CypA wt or CypA H126Q encoding vector were transfected with sgNS3/JFH1-Luc. Thereafter cells were treated with various concentrations of DEBIO-025 and 72 h later, cells were lysed and luciferase activity was determined. Naive Huh7-Lunet cells were analyzed in parallel. (A–C) Means and standard deviations of a representative experiment (2 independent repetitions) are shown.

To gain further insight into the role of CypA for replication of HCV, we wanted to take advantage of the fact that cyclosporine (CsA) binds to and sequesters CypA [4] and causes a profound inhibition of HCV replication [25],[38]. Moreover, we have recently shown that the cyclosporine analogue DEBIO-025 also inhibits replication of HCV, but the cellular target of this compound was not clear [5]. To clarify this question we first compared the antiviral activity of CsA and DEBIO-025 side-by-side in a JFH1 replicon system. Cells transfected with the sgNS3/JFH1-Luc replicon that expresses the firefly luciferase (Fig. 2A, upper panel) were treated 4 h post transfection for 72 h with various concentrations of CsA or DEBIO-025. Cells were then lysed and luciferase activity was determined. The JFH1 subgenome was potently inhibited both by CsA and DEBIO-025, with the latter having a higher antiviral activity (about 3–4 fold lower IC_50_ and IC_90_ (Fig. 5A)).

We next determined whether CypA is targeted by DEBIO-025 using transient replication assays with the same subgenomic JFH1 RNA. Transfected cells were treated with various concentrations of DEBIO-025 and after 72 h luciferase activity contained in cell lysates was determined. The results depicted in Fig. 5B show that HCV replication was much more sensitive to DEBIO-025 treatment in CypA knock-down cells whereas IC_50_ and IC_90_ values determined in CypB knock-down cells were comparable to naïve and control shRNA-transduced cells. Moreover, when dose-response assays were performed with CypA wild type ‘rescue’ cell lines, DEBIO-025 sensitivity was reduced even to beyond the level determined for naïve cells (Fig. 5C). This enhanced resistance is probably due to the elevated expression levels of CypA in the rescue cell line as compared to naïve cells. Most importantly, DEBIO-025 sensitivity was not affected by transduction of the gene containing the CypA isomerase mutation (H126Q) suggesting that an enzymatically active CypA is required for HCV RNA replication or that this CypA protein binds DEBIO-025 with lower efficiency. In summary, our results show that CypA is the target for the antiviral activity of DEBIO-025 and that isomerase activity of CypA appears to be required for HCV replication.

### A mutation in the C-terminus of NS5A confers DEBIO-025 resistance and reduces dependence of HCV RNA replication on CypA

Given the finding that DEBIO-025 can be used as a convenient tool for the pharmacological inhibition of CypA and to study its role in HCV RNA replication, we sought to identify the target of CypA in the HCV replicase by selection for DEBIO-025 resistance. Due to the high potency of DEBIO-025 and the small window between IC_50_ and IC_90_ (0.2 µg/ml and 0.5 µg/ml; Fig. 5A), attempts to select for resistance by using virus passage or cells persistently infected with Jc1 were not successful. Concentrations in the range of the IC_90_ or above lead to rapid elimination of HCV from cell cultures and treatment with a dose corresponding to the IC_50_ did not sufficiently affect replication. We therefore utilized a selectable JFH1-derived subgenome that carried a stable in-frame insertion of the red fluorescent protein (RFP) in domain 3 of NS5A [39], which allowed visualization of replicating HCV via RFP autofluorescence in live cells. Huh7-Lunet cells were transfected with this sgNS3/JFH1-neo/RFP replicon and cultured in double-selection medium containing 500 µg/ml of G418 and 0 or 0.5 or 1 µg/ml of DEBIO-025. After 18 cell passages, we had selected for a DEBIO-025 resistant cell pool with a calculated IC_50_ of about 1 µg/ml corresponding to an about 5-fold lower sensitivity as compared to non-selected cells (see Fig. 5A).

**Figure 6:**
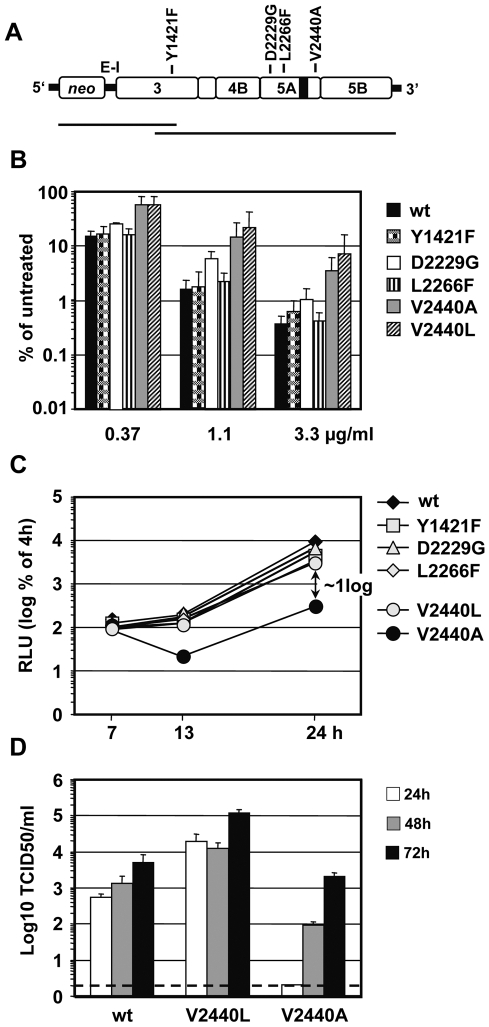
Identification and characterization of mutations conferring resistance against the antiviral activity of DEBIO-025.

To identify mutations that confer increased DEBIO-025 resistance and thus presumably reduced CypA dependence, replicons present in two independent RNA preparations of the cell pool were prepared from DEBIO-025 selected cells. From each RNA preparation the complete HCV coding region was amplified in two overlapping fragments (Fig. 6A) and 3 or 4 molecular clones of each fragment were subjected to nucleotide sequence analysis, respectively. As summarized in Table 1 and Fig. 6A, 4 mutations were identified residing in the NS3 helicase domain (Y1421F) or at various positions in NS5A. Two of these were mapped to domain 2 of NS5A (D2229G, L2266F) and one to the very C-terminus of domain 3 (V2440A). Quite surprisingly, we have recently identified position 2440, which is close to the NS5A-B cleavage site, as a major determinant of HCV assembly [40]. However, in case of this virus titer enhancing mutation (TEM) the substituting amino acid residue was leucine instead of the alanine found in the DEBIO-025 resistance selection.

**Table 1:** Mutations identified in JFH1/RFP replicons after selection for DEBIO-025 resistance.

HCV protein	Substitution frequency^a^	Amino acid substitution	Amino acid appearance^b^
NS3 helicase	7/7	Y 1421 F (391)	Y, F
NS5A domain II	5/7	D 2229 G (253)	D, E, A
NS5A domain II	7/7	L 2266 F (290)	E, L, R, H, M, D, N, G
NS5A domain III	7/7	V 2440 A (464)	V, A, I
NS5A domain III^c^	n.a.	V 2440 L (464)	V, A, I

a Number of DNA clones in which the mutation was found vs. number of clones that were analyzed. Note that all mutations were found with the 3′ PCR fragment (Fig. 6A) and therefore the identified mutations coexisted in the same replicon RNA.

b Amino acids found at the corresponding position of the polyprotein of HCV isolates available in the European HCV database and belonging to genotypes 1a,b,c; 2a,b,c,i,k; 3a,b,k; 4a,d,f; 5a; 6a,b,c,e,f,g,h,i,j, k,l,m,n,p; 7a.

c Virus titer enhancing mutation; data taken from [40].

n.a., not applicable.

Bracketed numbers refer to positions of the individual HCV protein of the JFH1 isolate.

Multiple sequence alignments covering HCV isolates of all genotypes as deposited in the European HCV database (euHCVdb) [41] revealed that all positions identified in DEBIO-025 selected replicons are polymorphic (Table 1). The mutations residing in the NS3 helicase and at the C-terminus of domain 3 of NS5A appear to be natural variants, because the substituting residues (phenylalanine in case of the helicase and alanine in case of NS5A) are present in patient isolates (Table 1). Other substituting residues are naturally found in case of the two mutations in domain 2 of NS5A.

The impact of these 4 selected mutations on DEBIO-025 sensitivity was determined by transferring each mutation individually into a wild type JFH1 luciferase replicon (sgNS3/JFH1-Luc; Fig. 2A) and determining replication fitness (Fig. 6C) as well as DEBIO-025 sensitivity (Fig. 6B). In this and all subsequent analyses we included the TEM V2440L, because it resides at the very same position like the putative resistance mutation V2440A. The results in Fig. 6B demonstrate that only the mutations affecting residue 2440 in NS5A reduced DEBIO-025 sensitivity to a significant extent. In fact, the IC_50_ of replicons containing the V2440A or the V2440L substitution was increased about 5- to 10-fold (Fig. 6B) whereas the mutations in domain 2 of NS5A had either minor or no effect. Replication fitness of only the V2440A mutant was reduced (about 10–15-fold as compared to the wild type) whereas all other mutants were unaffected (Fig. 6C).

Since V2440L is a TEM, we next assessed the impact of this and the V2440A substitution on virus production. The two mutations were introduced into the JFH1 wild type genome and virus titers released from cells at various time points after transfection were determined (Fig. 6D). In agreement with our earlier report [40] the V2440L substitution enhanced virus production without affecting RNA replication, whereas the V2440A substitution reduced virus production to an extent corresponding to the lower replication. These results suggest that only V2440L is a TEM.

**Figure 7:**
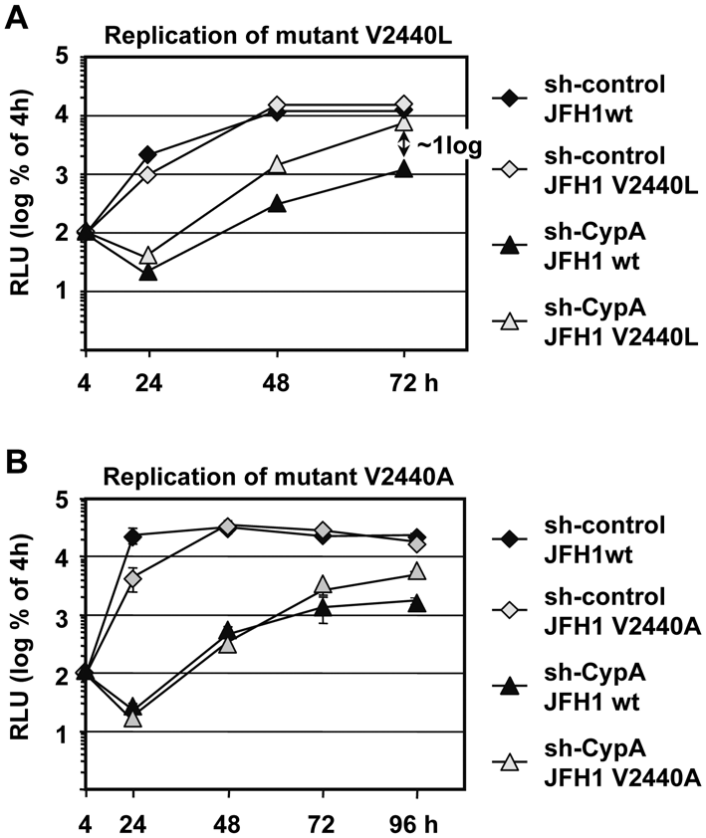
A mutation close to the NS5A/B cleavage site confers resistance to DEBIO-025 and renders HCV replication less dependent on CypA. (A) Replication of sgNS3/JFH1-Luc wt and the V2440L replicon in Huh7-Lunet cells with a stable knock-down of CypA or transduced with the retroviral vector control (sh-control). Cells were lysed at given time points after transfection with each of the replicon RNAs and luciferase activity in cell lysates was determined. Values were normalized to the 4 h value to exclude differences due to transfection efficiency. Note the higher replication of the V2440L mutant as compared with the wild type in CypA knock-down cells. (B) Experimental setup as in A, with the difference that the V2440A- and not the V2440L-mutant was used. Means and standard deviations of two independent experiments are shown in each panel.

The data described so far show that CypA is an important cellular determinant of HCV RNA replication and they suggest that DEBIO-025 blocks HCV replication by interfering with CypA. Having identified V2440L/A as DEBIO-025 resistance conferring mutations we assumed that they might render HCV replicons less dependent on CypA. To corroborate this assumption we performed transient replication assays with JFH1 wild type, JFH1 V2440L and JFH1 V2440A sgNS3-replicons in CypA knock down cells (Fig. 7A and B, respectively). In line with our assumption we found that replication of the V2440L mutant was less impaired in CypA depleted cells. The analogous result was found when we scored replication by immunofluorescence of transfected cells (not shown). In case of the V2440A mutation, due to reduced replication fitness only a slight but statistically significant difference (p<0.002, α = 0.05; for values 72 h and 96 h post transfection, respectively) compared to the wt replicon was apparent (Fig. 7B). These data suggest that the mutations close to the C-terminus of NS5A conferring DEBIO-025 resistance render HCV replication less dependent on CypA.

### Correlation between cleavage kinetic at the NS5A-B site and CypA dependence

**Figure 8:**
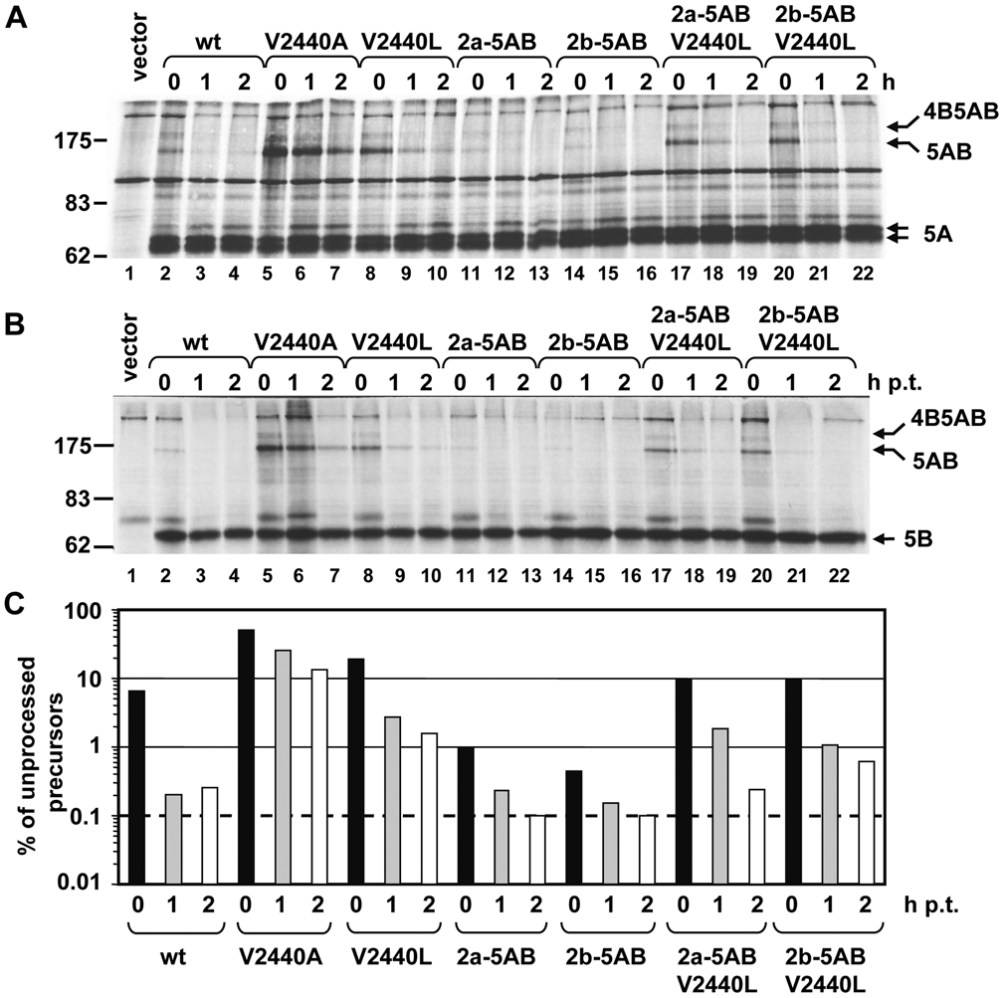
Alteration of the kinetics of polyprotein processing at the NS5A-B cleavage site by mutations conferring DEBIO-025 resistance. (A, B) Huh7-Lunet/T7 cells were transfected with subgenomic NS3 to NS5B JFH1 expression constructs derived from the wild type or mutants specified at the top of each panel or with the empty vector (pTM1-2; lane 1). After 24 h cells were pulse-labeled for 90 min with [^35^S] methionine/cysteine. Cells were lysed immediately (0) or incubated with non-radioactive medium for 1 or 2 additional hours. Immunoprecipitation was performed using either an NS5A- (A) or NS5B-specific antibody (B). Molecular weights are given on the left, arrows to the right point to the respective HCV proteins detected. (C) Amounts of unprocessed NS5AB and NS4B5AB precursors were quantified by phosphoimaging. Values obtained with the phosphoimage displayed in the upper panel are shown in panel C. Similar ratios were obtained with the phosphoimage of the NS5B-specific immunoprecipitation (data not shown).

We have recently shown that the V2440L substitution leads to delayed cleavage at the NS5A-B site [40]. Having identified another substitution at the same site that also confers DEBIO-025 resistance and lower dependence on CypA (V2440A), we wondered whether this mutation affects cleavage kinetics as well. We therefore performed pulse-chase experiments using a T7-based expression system. Huh7-Lunet cells stably expressing the T7 RNA polymerase were transfected with NS3 to NS5B polyprotein expression constructs corresponding to the wild type or containing the V2440L or the V2440A mutation. Proteins were radiolabeled metabolically with [^35^S] methionine/cysteine for 90 minutes and treated with non-radioactive medium for one or two hours. Cells were lysed and NS5A- and NS5B-containing proteins were isolated by immunoprecipitation with mono-specific antibodies (Fig. 8A and B, respectively; a quantification of the autoradiogram shown in panel A is given in panel C). We found that both mutations slowed down cleavage kinetics at the NS5A-B site, with the V2440A mutation causing a much stronger delay of cleavage than the leucine substitution (Fig. 8A, B, lanes 5–7). Best visible was the accumulation of an uncleaved NS5AB precursor protein as well as a precursor with an apparent molecular weight of about 175 kDa corresponding most likely to uncleaved NS4B5AB [17]. Only small amounts of these precursors were found in case of the wild type reflecting faster cleavage at the NS5A-B site. These results suggest a link between processing kinetic at this site and CypA dependence of HCV replication.

**Figure 9:**
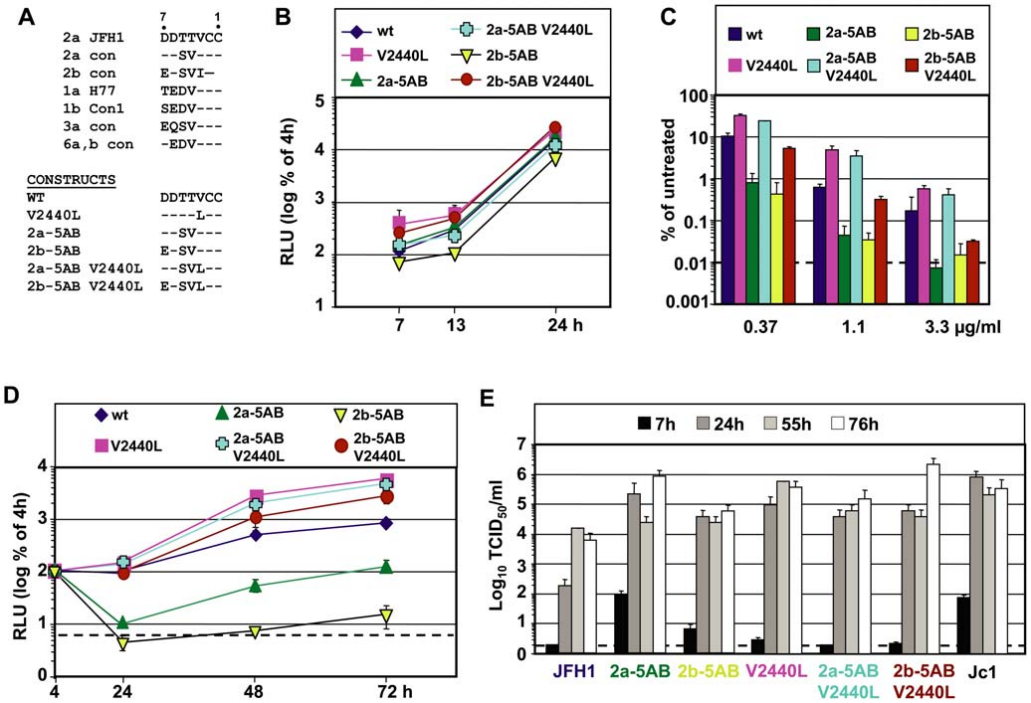
Properties of NS5A-B cleavage site mutants. (A) Alignment of amino acid consensus (con) sequences of various HCV genotypes or individual HCV isolates (H77, Con1, JFH1) at the P-side of the NS5A-B cleavage site. JFH1 (upper lane) was used as master sequence. Numbers at the top refer to the P-side positions. Amino acid sequences of cleavage site mutants are given in the lower part of this panel (CONSTRUCTS). (B) Time course of replication of subgenomic sgNS3/JFH1-Luc replicons specified in the top. Only the early time points up to 24 h post infection are shown. (C) Dose-response assay of cleavage site mutants. Subgenomic replicons were transfected into Huh7-Lunet cells and treated 4 h later with escalating concentrations of DEBIO-025. After 72 h cells were lysed and luciferase activity was determined. Values are normalized to transfected control cells that were mock treated. The means and standard deviations of two independent experiments are shown in panel B and C. (D) Replication kinetic of cleavage site mutants in Huh7-Lunet cells with a stable knock-down of CypA. Cells transfected with constructs specified in the top were lysed at given time points and luciferase activity was determined. Values were normalized for transfection efficiency as determined with the 4 h value. (E) Impact of NS5A-B cleavage site mutants on release of infectivity. Genomes depicted at the bottom of the panel were transfected into Huh7-Lunet cells and release of infectious HCV particles into the supernatant was determined by TCID_50_ assay at time points specified in the top. (D–E) The background of the assay is indicated with the horizontal dashed line. Means and standard deviations of a representative experiment are shown.

To substantiate this observation, we generated additional mutants affecting the NS5A-B cleavage site. Selection of these mutations was guided by a multiple alignment of amino acid residues at the P7-P1 positions of the NS5A-B cleavage site in HCV isolates deposited in the European HCV database (euHCVdb) [41] (Fig. 9A). Interestingly, amino acids at positions P7-P4 of the JFH1 isolate are rather unique amongst all the other genotypes including genotype 2 (Fig. 9A, upper half). In case of genotype 2a the P5 and P4 residues are serine and valine, respectively. Genotype 2b isolates have in addition a P7 glutamic acid residue and a P3 isoleucine residue. Based on this alignment we constructed two cleavage site mutants corresponding to the NS5A-B site of genotype 2a and (with the exception of the P3 isoleucine) to genotype 2b (Fig. 9A, lower half). In addition these mutations were combined with the P3 leucine substitution (V2440L) to determine its contribution on DEBIO-025 resistance, CypA dependence and cleavage kinetics in the context of the 2a and 2b cleavage site. As shown in Fig. 8, cleavage between NS5A and NS5B was enhanced with mutants 2a-5AB and 2b-5AB. A comparison of the protein pattern obtained after immunoprecipitation with the NS5A-specific antibody revealed that the NS5AB precursor was well detectable right after labeling in case of the wild type (Fig. 8A, lane 2) and this protein remained detectable throughout the chase period, albeit at low amounts (lane 3, 4). In case of the 2a and the 2b mutants the amounts of this precursor were lower after the 1 h labeling period and it was not detectable at later time points (lane 11–16). However, upon insertion of the V2440L substitution into each of these mutants, processing was again delayed compared to the wild type and cleavage kinetic was similar to the one observed with the V2440L single mutant (compare lanes 17–22 with 8–10 in Fig. 8A–B and quantification in Fig. 8C).

Upon transfection of Huh7-Lunet cells with replicons containing these mutations, we found that save for the 2b-5AB mutant, which was slightly impaired, replication of all the other mutants was well comparable to wild type (Fig. 9B). To our great surprise mutants 2a-5AB and 2b-5AB showed an about 10-fold increased DEBIO-025 sensitivity (Fig. 9C) and replicated less efficiently in stable CypA knock-down cells (Fig. 9D). However, the analogous mutants containing in addition the V2440L substitution were again less sensitive to DEBIO-025 (Fig. 9C) and replicated more efficiently in CypA knock-down cells (Fig. 9D). These results reveal a correlation between cleavage kinetics at the NS5A-B site, DEBIO-025 resistance and CypA dependence (Table 2). Delay of cleavage kinetics correlates with low DEBIO-025 sensitivity and reduced CypA dependence whereas ‘hyper-processing’ correlates with increased DEBIO-025 sensitivity and CypA dependence.

**Table 2:** Summary of phenotypes of NS5A mutants.

Mutant	Kinetic^1^	DEBIO-025 resistance	CypA dependence	Replication	Assembly^2^
V2440A	↓↓	↑	↓	↓	n.d.
V2440L	↓	↑	↓	u.a.	↑
2a-5AB	↑	↓	↑	u.a.	↑
2b-5AB	↑	↓	↑	u.a.	↑
2a-5AB V2440L	↓	↑	↓	u.a.	↑
2b-5AB V2440L	↓	↑	↓	u.a.	↑

1 Kinetic of cleavage at the NS5A-B site.

2 Production of infectious virus as deduced from TCID_50_ assay.

↓, lower than wild type.

↑, higher than wild type.

n.d., not determined.

u.a., unaffected.

Given the link between the V2440L mutation and enhanced HCV assembly, we tested all cleavage site mutations for their effect on virus production. As shown in Fig. 9E all cleavage site mutations increased virus titers and kinetics. With the exception of the 2b-5AB V2440L mutant, peak titers were reached already 24 h post transfection with all mutants and titers were elevated 100- to 1.000-fold. Especially for the 2a-5AB mutant, already 7 h after transfection infectious virus was detected in the culture supernatant (compare 2a-5AB also with the very efficient Jc1 in Fig. 9E). A significant, but less pronounced enhancement of kinetics and overall production of virus was achieved with mutant 2b-5AB. When the V2440L substitution was introduced into these mutants, kinetics were reduced, best visible with the 7 h values (Fig. 9E). In summary, our results reveal a correlation between cleavage kinetics at the NS5A-B site, DEBIO-025 resistance and CypA dependence. The data also suggest that cleavage kinetics at the NS5A-B site and virus assembly may be linked.

## Discussion

Exploitation of host cell machineries to achieve efficient replication is a strategy shared by all viruses. In case of HCV, Cyps have gained increasing interest because of the possibility to interfere pharmacologically with their activity thus providing a new approach for antiviral therapy [42]. In the present study we demonstrate that CypA but not CypB is the major player for HCV replication. This conclusion is supported by the profound impairment of replication by stable CypA, but not CypB knock-down and the rescue of viral replication by expression of CypA in these cells. Rescue was not achieved by expression of the active site mutant H126Q. This mutation resides in the hydrophobic pocket of CypA and causes a reduction of PPIase activity to about 1% of the wild type [36],[37]. Although this result argues for a role of PPIase activity in HCV replication, it should be kept in mind that CypA mutants that are impaired in isomerase activity have reduced substrate binding [37],[43]. Thus, PPIase activity and substrate binding cannot be separated genetically and therefore, the exact role of isomerase activity in HCV replication remains to be determined.

Cyps are a diverse group of proteins that share a PPIase activity and a 109 amino acids long Cyp-like domain [23]. There are 7 major Cyps in humans that differ in their N- and C-terminal sequences, which determine the intracellular localization of a given Cyp. For instance, CypA (as well as Cyp40 and CypNK) are cytosolic whereas CypB and CypC possess N-terminal signal sequences, which target the proteins to the ER-resident secretory machinery. CypD has a signal sequence recruiting the protein to mitochondria and CypE is localized in the nucleus. Although the role of Cyps in HCV replication has been established in several reports, conflicting results exist as to which individual Cyp is required. Two studies identified CypB, but not CypA, as the key player in HCV replication [24],[25]. One study describes that CypA, B and C are required [44] and the study by Yang and co-workers suggests that CypA, but not CypB and C is critical for HCV replication [26]. The results presented in this report are in full support of the work by Yang and colleagues. We also identify CypA as the key player in HCV replication and show that this host factor is the target of the cyclosporine analogue DEBIO-025. The reasons for the discrepant results is not known, but may in part be due to the use of different cell systems, HCV isolates or experimental conditions such as utilized siRNAs. However, we note that in our study HCV replication was impaired upon CypA knock-down both in Huh7-Lunet and Huh7.5 cells; two widely used Huh7-derived cell clones that are highly permissive for HCV replication. We also note that the impairment of HCV replication was found both with the JFH1 and the Con1 isolate similar to what Yang and colleagues described [26]. In support of these findings, a very recent study also describes CypA as the main actor in HCV replication [45]. Considering the subcellular localization, the cytosolic CypA is more likely to interact with the HCV replicase that also resides on the cytosolic side of the ER membrane, in contrast to the ER luminal CypB. In addition, CypB and C are expressed 10–150-fold lower in hepatoma cells as compared to CyPA thus increasing the likelihood of the latter to interact with the viral replicase [26].

In an attempt to study the mode-of-action of CypA, we wanted to exploit the possibility to interfere with this protein pharmacologically by using DEBIO-025, a compound that shows promise in clinical trials for the treatment of chronic hepatitis C [42]. The advantage of this compound is its higher anti-HCV activity as compared to CsA and reduced immunosuppressive activity, the latter being due to reduced calcineurin affinity [5],[46]. As shown here, dose-response assays with cell lines expressing various levels of CypA confirm that DEBIO-025 indeed targets this host cell factor: HCV replication can be sensitized to the antiviral activity of DEBIO-025 by stable knock-down of CypA whereas over-expression of CypA leads to partial DEBIO-025 resistance. Although these results confirm CypA as a DEBIO-025 target, it is not known whether other Cyps are also affected by this compound, because interference with these proteins (e.g. CypB) has no effect on HCV replication, which was used as a read-out in our assays.

By using replicons to select for DEBIO-025 resistance we identified Y1421F (Y391F in NS3) that resides in the NS3 helicase domain, two mutations in domain 2 of NS5A (D2229G/D253G; L2266F/L290F) and one mutation at the P3 cleavage site position of the NS5A-B junction (V2440A/V464A). Among these mutations, only V2440A (V464A in NS5A) reduced DEBIO-025 sensitivity and rendered HCV replication less dependent on CypA. We had recently identified a mutation at the very same site that enhances virus assembly about 100-fold without affecting RNA replication (V2440L/V464L) [40]. As shown here this mutation also confers DEBIO-025 resistance, renders HCV replication less dependent on CypA and slows down processing at the NS5A-B site. Results obtained with additional mutations at the NS5A-B site revealed a correlation between processing kinetics and CypA dependence (Table 2): mutants with slower cleavage kinetic require lower level of CypA and are less sensitive to DEBIO-025 treatment compared to wild type whereas mutants with accelerated cleavage kinetic are more dependent on CypA and more sensitive to DEBIO-025 treatment. So far we can only speculate about the underlying mechanism, but for any model the following observations should be considered:

1. CypA and CypB are PPIases which appear to bind to the C-terminal region of NS5B [24],[26];
2. CypA and/or CypB may induce a conformational change in NS5B by acting on a critical proline residue in the C-terminal region of NS5B [24];
3. Based on the X-ray crystal structure of NS5B, the Cyp binding site appears to be poorly accessible for protein-protein interaction in the folded NS5B molecule and may only be accessible in the unfolded protein, e.g. during protein synthesis (reviewed in [47]);
4. Cleavage at the NS5A-B site occurs rapidly and NS5A-B precursors are present in very low amounts [17],[18].

One possible explanation for our observations is that the CypA binding site in the C-terminal region of NS5B might be accessible only in an extended (non-folded) conformation right after protein synthesis. Proper folding of NS5B may require liberation of the N-terminus from the polyprotein (i.e. cleavage at the NS5A-B site). NS5B folded in the absence of CypA may adopt a conformation that is enzymatically inactive or that is not competent to be incorporated into the replicase complex [48]. In this model binding of CypA to NS5B would be required to induce a conformation necessary for replicase formation or activity. Binding however, would be possible only during a short time period, i.e. prior to or shortly after release of full length NS5B from the polyprotein. In case of a delayed processing (V2440L/A) the time during which the CypA binding site is accessible is extended and therefore lower amounts of CypA would suffice to bind to NS5B. Nevertheless, also under these circumstances CypA is required for HCV replication thus explaining why these replicons are still susceptible to DEBIO-025, albeit to a lesser extent. In case of hyper-processing the time for CypA binding would be much shorter and therefore the chance for interaction with NS5B would be lower. This can however be compensated by higher CypA amounts. This model would also explain why the mutations modulating NS5A-B processing do not have an effect on RNA replication in naïve cells. Under normal conditions, CypA levels are not limiting and even in case of the hyper-processing mutants CypA amounts are sufficient to bind to NS5B. Only when CypA levels are reduced either by knock-down of CypA expression or by DEBIO-025 treatment the chance that CypA can bind to NS5B during the much shorter time period, compared to wild type, becomes apparent. In contrast, delaying processing will extend the time for CypA binding and therefore even lower CypA amounts would be sufficient for binding to NS5B. However, a too slow processing at the NS5A-B site appears to impair replicase activity as deduced from the reduced replication of the V2440A mutant.

The CsA resistance mutations residing in domain 2 of NS5A that have been described in a recent study [27] may affect NS5B activity indirectly by altering binding of NS5A to NS5B. It has been shown that NS5A binds to NS5B and this interaction appears to modulate RdRp activity [49]. Alternatively, these mutations may affect directly a function of NS5A required for RNA replication [50] or polyprotein cleavage kinetics, but these possibilities need to be addressed. Finally, the impact of altered cleavage kinetics on HCV assembly might be an epiphenomenon caused by the mutations in domain 3 of NS5A itself that is a major determinant of virus assembly [15],[16].

Another surprising observation we made is the higher sensitivity of replication of JFH1 full length genomes towards CypA depletion and the impairment of virus production. This observation suggests that CypA may have an additional target outside of the minimal replicase. The most likely candidate is NS2, because it contributes indirectly to RNA replication and is required for assembly [12],[13] whereas all other proteins residing in the N-terminal domain of the polyprotein (core, E1, E2, p7) are completely dispensable for RNA replication. In fact, the stronger inhibition observed with the JFH1 sgNS2-replicon containing NS2 and the impairment of HCV assembly supports this assumption. Since NS2 contributes to RNA replication indirectly by cleaving off itself from NS3, a possible explanation is that CypA supports proper folding of NS2. However, in addition to a viral factor CypA may also be required for host cell factor(s) contributing to virus production. In fact, preliminary results suggest that CypA may be required for integrity of lipid droplets (A. Kaul, C. Berger, and R. Bartenschlager, unpublished observation), which play a key role in HCV assembly [20]–[22]. Further studies will be required to identify CypA-dependent viral and host cell factors required for HCV RNA replication, assembly and eventually egress.

## Materials and Methods

### Cell culture and cell lines

All cell lines were grown in Dulbecco's modified minimal essential medium (DMEM; Life Technologies, Germany) supplemented with 2 mM L-glutamine, nonessential amino acids, 100 U/ml of penicillin, 100 µg/ml of streptomycin, and 10% fetal calf serum. The experiments were performed either in Huh7-Lunet cells supporting high level RNA replication or Huh7.5 cells that are highly infectable [51],[52]. For selection of DEBIO-025 resistant JFH1 replicons Huh7-Lunet sgNS3/JFH1-neo/RFP replicon cells [39] were double-selected for 18 cell passages with 500 µg/ml of G418 and 0 or 0.5 or 1 µg/ml of DEBIO-025. For transient dose response experiments subgenomic Luc-JFH1 replicons were transfected into Huh7-Lunet cells and 4 h post electroporation cells were treated with various concentrations of Cyclosporine A (CsA) or DEBIO-025, respectively. Seventy two hours later the impact on replication was determined by luciferase assay.

### Plasmid construction

All nucleotide and amino acid numbers refer to the JFH1 genome (GenBank accession no. AB047639) or the Con1 genome (GenBank accession number AJ238799). The chimera Jc1-Luc was described recently [33]. For replication analyses the subgenomic reporter replicon pFKI_389_Luc/NS3-3′_dg_JFH (abbreviated as sgNS3/JFH1-Luc) and the replication deficient mutant carrying a deletion of the NS5B active site (sg/luc/JFH1/ΔGDD) were used [39]. The basic Con1 construct pFK-rep PI-luc/ET has been described somewhere else [31]. To generate the subgenomic JFH1 replicon construct pFKI_389_Luc/NS2-3′_dg_(designated sgNS2/JFH1-Luc in this report) we performed two separate PCRs: the first one with sense primer S/EMCV190 (5′-AATGCAAGGTCTGTTGAATGT-3′) and antisense primer A/EMCV-NS2 (5′-CGTGCACAGGTGCGTCATACATGGTATCATCGTGTTTTTCA-3′) and pFKI_389_Luc/NS3-3′_dg_[39] as template; the second one with primers A/JFH1/2894 (5′-TGACGGCCCACGCGATGCCAT-3′) and S/EMCV-NS2 (5′-TGAAAAACACGATGATACCATGTATGACGCACCTGTGCACG-3′) and pFKI_389_Luc/Core-3′_dg_JFH [32] as template. Fragments were combined by overlap-PCR using S/EMCV190 and A/JFH1/2894 and the resulting DNA fragment was inserted into pFKI_389_Luc/Core-3′/DelE1E2_dg_JFH [32]. The final construct pFKI_389_Luc/NS2-3′_dg_JFH contains (5′ to 3′) the T7 promoter sequence fused to nucleotides 1 to 389 of the JFH1 consensus sequence, the firefly luciferase gene, the encephalomyocarditis virus (EMCV) IRES, the NS2 to 5B coding sequence, the 3′ NTR of JFH1, the hepatitis delta virus genomic ribozyme (dg) and the T7 terminator sequence. Amino acid substitutions were introduced by PCR-based site-directed mutagenesis and amplified DNA fragments were analyzed by automated nucleotide sequencing using an ABI 310 sequencer (Applied Biosystems, Darmstadt, Germany).

### Generation of stable knock-down and rescue cell lines

For CypA and CypB knock-down microRNA-based shRNA lentiviral vectors were produced by co-transfecting 293T cells with transfer vectors encoding the puromycin resistance gene and a shRNA targeting CypA, CypB, or a control shRNA targeting luciferase, the HIV-1 packaging plasmid psPAX2, and a VSVg expression plasmid (pMD2.G) using Lipofectamine 2000 (Invitrogen). The shRNA targeting sequences were: luciferase, 5′-tacaaacgctctcatcgacaag-3′ (not present in the luciferase gene of the reporter replicon and reporter virus), CypA, 5′-ctggattgcagagttaagttta-3′; and CypB, 5′-gccgggtgatctttggtctctt-3′. Transfection medium was changed the next day and viral supernatant was harvested 48 hrs after transfection, clarified by centrifugation (5 min at 200×g), and filtered through a 0.45 µm syringe filter (Sarstedt). Huh7-Lunet and Huh7.5 cells were transduced on two consecutive days and placed into selection medium containing 5 µg/ml puromycin (Sigma) 72 h after transduction. For selected Huh7-Lunet cell pools, knock-down of CypA and CypB was stable for at least 20 passages and cell growth as well as viability was not affected (long-term passage of Huh7.5 cell pools was not performed). For stable over-expression of CypA in CypA knock-down cells we used a lentiviral vector system. 293T cells (5×10^5^) were seeded in each well of a 6-well cell culture plate in complete DMEM. About 24 h later, cells were transfected with lentiviral plasmids by using Lipofectamine 2000 or Lipofectamine LTX/Plus Reagent (Life Technologies) according to the instructions of the manufacturer. To generate the CypA rescue cell lines, 2.5 µg of the transfer vector pWPI-CypAwt or pWPI-CypA/H126Q, was transfected together with 2 µg of the packaging vector (pCMV) and 0.6 µg of the VSV envelope vector (pMD.G) into 293T cells by lipofection as described above. After 16–24 h medium (2 ml) was replaced by fresh one and an additional 24 h later culture supernatant was filtered through 0.45 µM pore size polyvinylidene difluoride (PVDF) syringe filter (Carl Roth GmbH, Karlsruhe Germany) and used for transduction. Huh7-Lunet/CypA knock-down cells were seeded at a density of 1–2×10^4^ cells per well of a 12-well plate and inoculated with 0.5–1 ml of filtered supernatant. Twenty four hours later, inoculation was repeated. Two to three days after transduction medium was replaced by fresh cell culture medium containing puromycin and/or blasticidin S. Drug concentrations were increased steadily during cell passages up to 10 µg/ml puromycin in case of Huh7-Lunet or 20 µg/ml in case of Huh7.5 and 20 µg/ml blasticidin S (Life Technologies).

### In vitro transcription and electroporation of HCV RNAs

In vitro transcription and electroporation of HCV RNAs was performed as described previously [40]. Transfected cells were immediately diluted into complete DMEM and seeded as required for the given assay.

### Preparation of total RNA, amplification of replicon RNA by RT-PCR and cloning of amplified DNA fragments

Total RNA was isolated from a confluent 6 cm diameter dish of Huh7-Lunet cells containing the sgJFH1-neo/RFP replicon by using the Nucleo Spin RNAII Kit (Macherey-Nagel, Düren, Germany) as recommended by the manufacturer. One µg total RNA and 50 pmol of primer A9482 (5′-GGA ACA GTT AGC TAT GGA GTG TAC C-3′) were used for cDNA synthesis by using the Expand-RT system (Roche, Mannheim, Germany) as recommended by the manufacturer. Two to four microliters of the reaction mixture were used to amplify the complete open reading frame in two overlapping fragments with the Expand Long Template PCR kit (Roche) according to the instructions of the manufacturer. To amplify the 5′ half of the replicon, PCR was performed with primers S59-EcoRI (5′-TGT CTT CAC GCA GAA AGC GCC TAG-3′) and A4614 (5′-CTG AGC TGG TAT TAT GGA GAC GTC C-3′) and the PCR product was inserted into sgJFH1-Luc after restriction with EcoRI and SpeI. The 3′ half of the HCV genome was amplified with primers S3813 (5′-GGA CAA GCG GGG AGC ATT GCT CTC-3′) and A9466-MluI (5′-AGC TAT GGA GTG TAC CTA GTG TGT GCC-3′) and after restriction with SpeI and MluI, the fragment was inserted into pFK-I_389_neo/NS3-3′/Con [35]. Sequence analysis was performed with a set of primers covering the complete replicon sequence.

### Indirect immunofluorescence

Huh7-Lunet and Huh7.5 derived cell lines were seeded onto glass cover slips in 24-well plates at a density of 2–4×10^4^ cells per well. Three days after seeding cells were fixed by treatment with methanol at −20°C for 10 min., washed 3 times with PBS, incubated for 30 min in 5% normal goat serum or bovine serum albumin (diluted in PBS) and incubated for 1 h at room temperature (RT) with one of the following primary antibodies: antiCyPA rabbit polyclonal antiserum (BIOMOL International, LP) diluted 1∶400, or antiCypB rabbit polyclonal antiserum (Affinity Bio Reagents) diluted 1∶800, or antiNS5A mouse monoclonal antibody (9E10) [53]. After an extensive wash with PBS, cells were treated with Alexa Fluor 488 or 546 conjugated antibodies, targeting rabbit or mouse IgG domains (dilution 1∶1,000 in normal goat serum) for 1 h at RT in complete darkness. Unbound secondary antibodies were removed by washing three times with PBS and once with water. DNA was stained with 4′, 6′-diamidino-2-phenylindole (DAPI; Molecular Probes, Karlsruhe, Germany) for 1 min at RT. Finally samples were mounted on slides with FluormountG (Southern Biotechnology Associates, Birmingham, USA) and analyzed by using fluorescence microscopy. For quantitation of immunofluorescence signals raw pictures were imported into the image J software package and pixel intensities based on 8-bit gray value were determined using the histogram function of the program (National Institutes of Health, Bethesda, MD, USA).

### Luciferase assays

Quantification of luciferase reporter activity was used to determine transient HCV RNA replication as described previously [33]. In brief, transfected Huh7-Lunet cells were resuspended in 41 ml complete DMEM and 1.5 ml of the suspension was seeded in each well of a 12-well plate. For each time point, duplicates of wells were harvested. For dose response experiments, 4 h post electroporation transfected cells were treated in duplicate with different concentrations of cyclosporine A (CsA) or DEBIO-025 and analyzed 72 h later by luciferase assay. Cells were washed once with PBS, 350 µl of lysis buffer (0.1% Triton X-100, 25 mM glycylglycine, 15 mM MgSO_4_, 4 mM EGTA and 1 mM DTT, pH 7.8) was added and freeze-thaw lysates were prepared. For each well, two times 100 µl lysate was mixed with 360 µl assay buffer (25 mM glycylglycine, 15 mM MgSO_4_, 4 mM EGTA, 1 mM DTT, 2 mM ATP and 15 mM K_2_PO_4_, pH 7.8) and, after addition of 200 µl of a luciferin solution (200 µM luciferin, 25 mM glycylglycine, pH 8.0), measured for 20 s in a luminometer (Lumat LB9507; Berthold, Freiburg, Germany). Kinetic of replication was determined by normalizing the relative light units (RLU) of the different time points to the respective 4 h value. For dose response experiments the RLU obtained with cyclosporine A (CsA) or DEBIO-025 treated cells were normalized to the corresponding values obtained with untreated cells.

### Metabolic radiolabeling of proteins and immunoprecipitation

A total of 2.5×10^5^ Huh7-Lunet/T7 cells [54] were seeded in each well of a 6-well cell culture plate in complete DMEM. About 24 h later, cells were transfected with 2.5 µg per well pTMNS3-3′JFH1 wild type or analogous constructs containing mutations specified in the results section or empty vector (pTM1-2) [55]. Transfection was performed by using Lipofectamine LTX/Plus Reagent (Life Technologies) according to the instructions of the manufacturer. After 4 h, cells were washed once with methionine/cysteine-free medium and starved for 1 h. For radiolabeling cells were incubated for 90 min in 1 ml methionine/cysteine-free medium, supplemented with 2 mM glutamine, 10 mM Hepes (pH 7.5), and 150 µCi/ml of Express Protein labeling mix (Perkin Elmer, Boston). Cells were lysed either directly or washed with PBS and incubated in complete DMEM for 1 or 2 h. Cell lysates were prepared by using NPB (50 mM Tris-Cl [pH 7.5], 150 mM NaCl, 1% Nonidet P-40, 1% sodium deoxycholate, 0.1% SDS, 1/10,000 vol aprotinin (1 U/ml), 1/1,000 vol leupeptin (4 mg/ml) and 1/100 vol phenyl-methyl-sulfonyl-fluoride (100 mM)) and cleared by centrifugation at 13,000 g for 15 min at 4°C. Cleared lysates were used for immunoprecipitation using either the NS5A-specific monoclonal antibody 9E10 [53] or a polyclonal NS5B-specific antibody. Immunocomplexes were dissolved in 70 µl 2× sample buffer (400 mM Tris pH 8.8, 10 mM EDTA, 0.2% bromophenolblue, 20% sucrose, 3% SDS and 2% ß-mercaptoethanol), separated in a 10% polyacrylamide-SDS gel and analyzed by autoradiography. HCV-specific bands were quantified by phosphoimaging using the QuantityOne software (BioRad, Munich, Germany).

### Western blot analysis

Huh7-Lunet cells of a confluent 6-well cell culture plate were washed once with ice-cold PBS and lysed with NP40 buffer buffer (50 mM Tris-HCl pH 8, 150 mM NaCl, 1% NP40). After 30 min incubation at 4°C, cell debris was pelleted by centrifugation for 30 min at 10.000 g and at 4°C. Four µg of cleared supernatant was diluted in 2× sample buffer, heated 5 min at 95°C and loaded onto a 12.5% polyacrylamide-SDS gel. After electrophoresis proteins were transferred to a PVDF membrane (PerkinElmer Life Sciences) for 1 h with an electric current of 1 mA/cm^2^. Membrane was blocked in PBS supplemented with 0.5% Tween (PBS-T) and 5% dried milk (PBS-TM) for at least 1 h prior to 1 h incubation with primary antibody diluted 1∶1,000 in PBS-TM. Membrane was washed 3 times with PBS-T and incubated for 1 h with horseradish-peroxidase conjugated anti rabbit secondary antibody diluted 1∶10,000 in PBS-TM. Bound antibodies were detected after 3 times washing with the ECL Plus Western Blotting Detection System (GE Healthcare Europe, Freiburg, Germany).

### Quantitation of HCV core protein

HCV core protein in transfected cells or cell culture supernatants was quantified using the Ortho® trak-C™ ELISA kit (Ortho Clinical Diagnostics, Neckargemünd, Germany). Lysates of transfected Huh7-Lunet cells were prepared by addition of 1 ml per 6 cm diameter culture dish of PBS containing 1% Triton X-100, 1/10,000 vol aprotinin (1 U/ml), 1/1,000 vol leupeptin (4 mg/ml) and 1/100 vol phenyl-methyl-sulfonyl-fluoride (100 mM). Lysates were cleared by centrifugation (18,000×g; 5 min) and samples were diluted 1∶10 or higher and processed for ELISA according to the manufacturer's protocol. Culture supernatants were filtered through 0.45 µm pore-size filters and used directly for core ELISA. Colorimetric measurements were performed using a Sunrise colorimeter (Tecan Trading AG, Switzerland). Kinetic of replication was determined by normalizing the intracellular core amount of the different time points to the respective 4 h value. To determine the efficiency of core protein release, the percentage of extracellular core to total core protein (the sum of intra- and extracellular core protein) was calculated.

### Determination of virus titers in cell culture supernatants

Virus titers were determined as described elsewhere with slight modifications [52]. Huh7.5 target cells were seeded at a concentration of 1.1×10^4^ cells per well of a 96-well plate in a total volume of 200 µl complete DMEM. Twenty four hours later, serial dilutions of virus containing supernatant were added with 6 wells per dilution. Three days later, cells were washed with PBS and fixed for 20 min with ice-cold methanol at -20°C. After three washes with PBS NS5A was detected with a 1∶2,000 dilution of antibody 9E10 in PBS for 1 h at room temperature. Alternatively NS3 was detected with a 1∶100 dilution of antibody 2E3 (kindly provided by H. Tang, Florida State University, USA) in PBS for 1 h at RT. Cells were washed again three times with PBS and bound primary antibodies were detected by incubation with peroxidase – conjugated or Alexa Fluor 546 – conjugated anti mouse antibody (Sigma – Aldrich), respectively, diluted 1∶1,000 in PBS. After 1 h incubation at room temperature cells were washed three times with PBS and in case of peroxidase – conjugated antibody the Vector NovaRED substrate kit (Linaris Biologische Produkte GmbH, Wertheim, Germany) was used for detection. Virus titres (50% tissue culture infective dose per ml (TCID_50_/ml)) were calculated based on the method of Spearman and Kärber [56],[57].

## Supporting Information

Figure S1 Impact of stable CypA or CypB knock-down on Dengue virus RNA replication. Huh7-Lunet cells (left panel) and Huh7.5 cells (right panel) either stably transduced with the shRNA vector (sh-control) or a vector encoding the CypA- or CypB-specific shRNA or naive cells were transfected with a subgenomic luciferase reporter replicon derived from the Dengue virus 2 isolate NGC (shown at the top). Cells were lysed at given time points after transfection, and luciferase activity in cell lysates was determined. Means and standard deviations of a representative experiment are shown. Details of this replicon will be described elsewhere. Cap, RNA cap structure; luc, firefly luciferase; ubi, ubiquitin.
(0.34 MB TIF)
