# Correction: Essential Role of Cyclophilin A for Hepatitis C Virus Replication and Virus Production and Possible Link to Polyprotein Cleavage Kinetics

**DOI:** 10.1371/annotation/29dffb2a-5d37-4196-b166-b0b15bc2f3fd

**Published:** 2009-09-01

**Authors:** Artur Kaul, Sarah Stauffer, Carola Berger, Thomas Pertel, Jennifer Schmitt, Stephanie Kallis, Margarita Zayas, Volker Lohmann, Jeremy Luban, Ralf Bartenschlager

The seventh author's name was incorrect. The correct name is Margarita Zayas. 

