# Supplementary figures and images for: Essential Role of Cyclophilin A for Hepatitis C Virus Replication and Virus Production and Possible Link to Polyprotein Cleavage Kinetics
Source: PLoS Pathog. 2009 Aug 14;5(8):e1000546. doi: 10.1371/journal.ppat.1000546 (PMC2718831; doi:10.1371/journal.ppat.1000546)

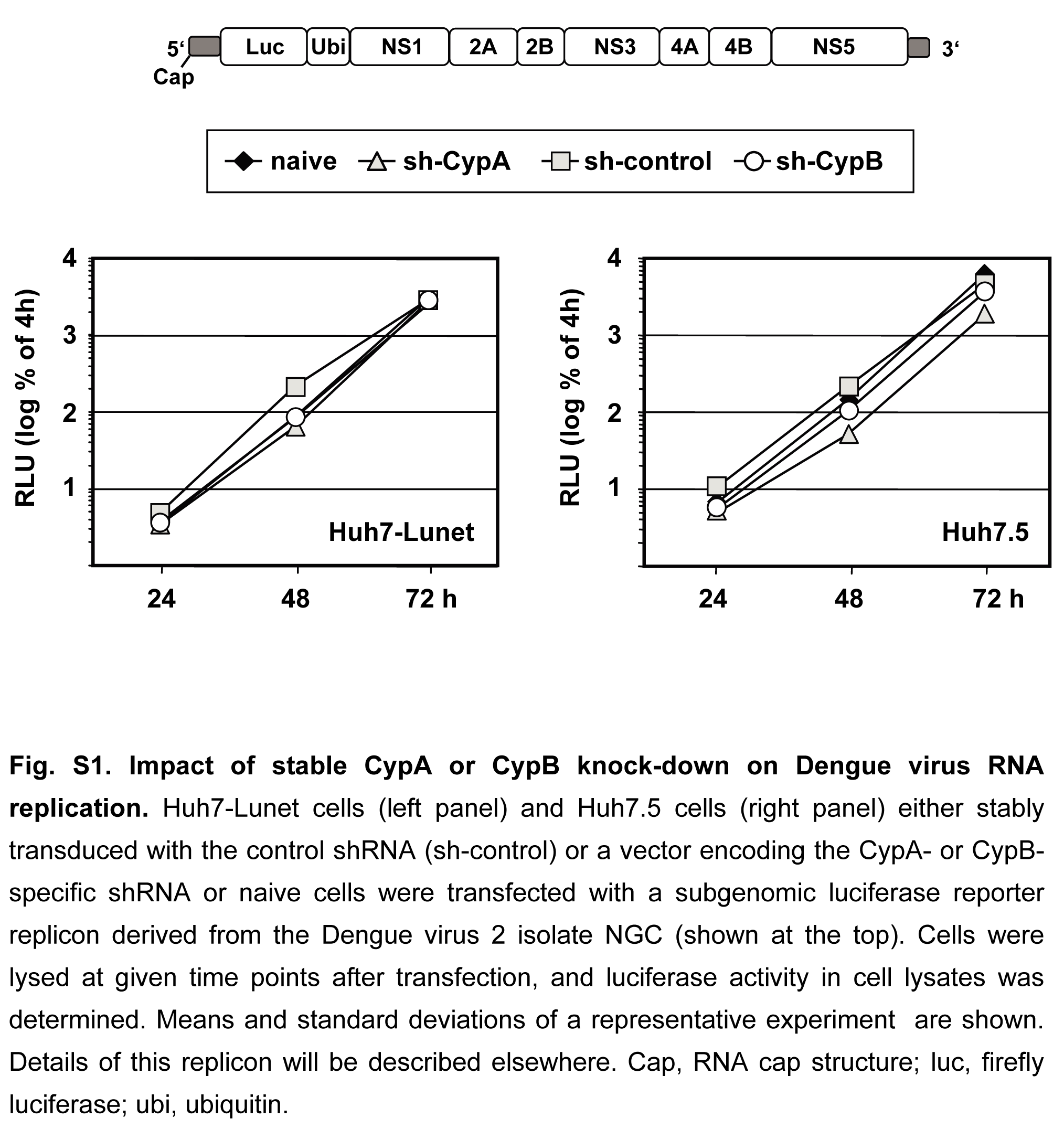

Supplement: Figure S1 — Impact of stable CypA or CypB knock-down on Dengue virus RNA replication. Huh7-Lunet cells (left panel) and Huh7.5 cells (right panel) either stably transduced with the shRNA vector (sh-control) or a vector encoding the CypA- or CypB-specific shRNA or naive cells were transfected with a subgenomic luciferase reporter replicon derived from the Dengue virus 2 isolate NGC (shown at the top). Cells were lysed at given time points after transfection, and luciferase activity in cell lysates was determined. Means and standard deviations of a representative experiment are shown. Details of this replicon will be described elsewhere. Cap, RNA cap structure; luc, firefly luciferase; ubi, ubiquitin. (0.34 MB TIF) [file ppat.1000546.s001.tif]
